# Vitamin D and Influenza—Prevention or Therapy?

**DOI:** 10.3390/ijms19082419

**Published:** 2018-08-16

**Authors:** Beata M. Gruber-Bzura

**Affiliations:** Department of Biochemistry and Biopharmaceuticals, National Medicines Institute, Str. Chelmska 30/34, 00-725 Warsaw, Poland; b.gruber@nil.gov.pl; Tel.: +22-841-39-91; Fax: +22-841-06-52

**Keywords:** vitamin D, influenza, respiratory tract infections

## Abstract

Vitamin D generates many extraskeletal effects due to the vitamin D receptor (VDR) which is present in most tissues throughout the body. The possible role of vitamin D in infections is implied from its impact on the innate and adaptive immune responses. A significant effect is also the suppression of inflammatory processes. Because vitamin D could be acknowledged as a “seasonal stimulus”, as defined by R. Edgar Hope-Simpson, it would be crucial to prove it from a potential easy and cheap prophylaxis or therapy support perspective as far as influenza infections are concerned. The survey of the literature data generates some controversies and doubts about the possible role of vitamin D in the prevention of influenza virus. The most important point is to realise that the broad spectrum of this vitamin’s activity does not exclude such a possibility. According to most of the authors, more randomized controlled trials with effective, large populations are needed to explore the preventive effect of vitamin D supplementation on viral influenza infections.

## 1. Introduction

The popularity of vitamin D as a vitamin with a broad spectrum of activity is still growing. There are many papers published each year about its properties, including ensuring our bone health. Now, we know that vitamin D is associated with cancer, diabetes, cardiac, and gastrointestinal diseases, and, most interestingly, even with events of unknown etiology, such as inflammatory bowel disease [1]. The phenomenon of its multidirectional activity is possibly due to the presence of the VDR in most nonskeletal human cells. New perspectives on vitamin D activity and targets are very important from, of course, the scientific point of view, but, and this is crucial, because of increasing awareness of the deficiency of this vitamin in different populations across the world and the need for its supplementation.

Through the VDR, vitamin D can also modulate the innate and acquired immune system. This has prompted the idea of exploring the impact of vitamin D on the efficacy of our immune system in fighting off difficult-to-treat viral infections, such as influenza, especially because reaching an optimal level of this “medicine” is cheap and easy for everybody.

The aim of this paper was to present recent data on the possible role of vitamin D in modulating the immune response in influenza virus infection and its potential preventive and therapeutic potential in that disease.

This review is based on an electronic search of articles in the PubMed database, including papers published mostly in the last eight years up until 2018 in that field. The relevant papers are also included. All research articles were found with a combination of the following keywords: vitamin D and influenza, vitamin D and respiratory illnesses, vitamin D and influenza vaccines, and vitamin D and infections. Published articles included in this meta-analysis were selected on the basis of the following criteria: they must have been published in English or Polish, concerning the association between the immune response and vitamin D serum concentration or supplementation, and defining the type of trial, the number of participants, outcome measure, and including statistical analysis.

## 2. The Metabolism, Action, and Guidance Serum Concentration of Vitamin D

### 2.1. Metabolism

**Figure 1:**
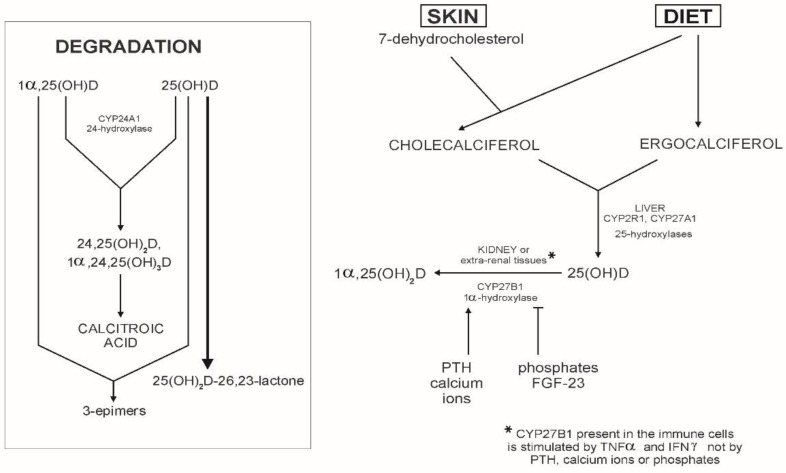
Metabolic pathways of vitamin D. Abbreviations: CYP24A1 (cytochrome P450-associated 24-hydroxylase); CYP2R1 and CYP27A1 (cytochrome P450-associated 25-hydroxylases); CYP27B1 (cytochrome P450-associated 25(OH)D3-1α-hydroxylase); PTH (parathormone); FGF-23 (fibroblast growth factor-23); TNFα (tumour necrosis factor α); IFNγ (interferon γ).

Vitamin D comes from two sources: skin synthesis from the precursor—7-dehydrocholesterol—to cholecalciferol upon UVB radiation, and from the diet as cholecalciferol (D_3_) or ergocalciferol (D_2_). Metabolic pathways (Figure 1), common for both forms, include: 25-hydroxylation to calcidiol (25(OH)D), which is carried by the liver enzymes CYP2R1 and CYP27A1 (cytochrome P450-associated 25-hydroxylases), followed by 1α-hydroxylation to the active metabolite 1α,25–dihydroxyvitamin D_3_ (calcitriol, 1α,25(OH)_2_D), catalysed by cytochrome P450-associated 25(OH)D(3)-1α-hydroxylase (CYP27B1), the enzyme present in the kidney but also in other extrarenal tissues, including immune cells [2,3,4]. Due to a developed feedback loop system, the metabolic activation of chole- and ergocalciferol and catabolic reactions are strictly regulated. The positive regulators of 1α,25(OH)_2_D production are parathormone (PTH), secreted by parathyroid glands, and calcium level, and the negative ones are phosphate level and fibroblast growth factor-23 (FGF-23). All of them affect the activity of 1α-hydroxylase [5].

Unlike the renal form, CYP27B1 present in the immune cells is not regulated by PTH, FGF-23, calcium, or phosphate signaling, but is stimulated by cytokines such as tumour necrosis factor α (TNF α) and interferon (IFNγ) [4,6]. In turn, CYP27B1 in keratinocytes is upregulated in response to injury and Toll-like receptor (TLR) activation [4]. Extrarenal expression of that enzyme may also be promoted by alternate pathogen recognition receptors (PRRs) [7]. Regulation of extrarenal 1α-hydroxylase is strongly dependent on the concentration of the circulating 25(OH)D [5].

Both metabolites, 25(OH)D and 1α,25(OH)D, are transported in the bloodstream by the carrier vitamin D binding protein (DBP), with higher affinity of 25(OH)D to that transporter [5]. Degradation of both metabolites is catalysed by CYP24A1, a multicatalytic enzyme that results in 24-hydroxylation and the formation of 24,25(OH)_2_D_,_ and 1α,24,25(OH)_2_D, which are subsequently converted to calcitroic acid [2,5]. The circulating calcidiol may be also converted by CYP24A1 to the inactive 25(OH)_2_D-26,23-lactone and 24,25(OH)_2_D [2].

Some metabolites, such as 3-epimers, which are formed by epimerization of the C-3 in ring A of 25(OH)D, 1α,25(OH)_2_D, and 24,25(OH)_2_D, have only slightly weaker biological activity than 1α,25(OH)_2_D. Such epimers were first reported in human keratinocytes in 1994 [8].

### 2.2. Nongenomic and Genomic Action

Vitamin D affects cellular metabolism by genomic and nongenomic pathways. Mostly, it acts through binding with VDR, and then, after forming a heterodimer with the retinoid X receptor (RXR) and translocating to the nucleus, binds to vitamin D responsive elements (VDRE) in DNA and controls gene transcription.

The nongenomic pathway includes rapid reactions at the cell membranes and has been reported for thyroid hormone, estrogen, and corticosteroids [5,9,10,11]. Probably via a membrane-bound receptor protein (1α,25(OH)_2_D membrane-associated rapid response steroid-binding protein, also known as endoplasmic reticulum stress protein 57), vitamin D regulates rapid cellular calcium efflux and calcium-activated chloride channel activity [11].

Nongenomic responses to the active vitamin D metabolite continue via the messenger systems of phospholipase C, protein kinase C, and phosphatidylinositol-3’-kinase (PI3K), initiating the opening of calcium channels and Ras/MAPK signal transduction [7].

Beyond the role in calcium homeostasis and bone metabolism, vitamin D generates many extraskeletal effects through the VDR, which is present in most tissues throughout the body [11,12]. Affinity to the VDR is much higher for 1α,25(OH)_2_D (K_a_ = 10^−10^ M) than for 25(OH)D (K_a_ = 10^−8^ M) [12]. Activation of that receptor is the basis for the regulation of approximately 3% of the human genome, affecting immune and neurological functions and playing a role in skin, cardiovascular, and gastrointestinal diseases, cancer inhibition, or inhibition of autoimmune diseases [5,10].

As was found in recent years, polymorphism in some enzymes and proteins related to vitamin D, such as DBP, CYP28B1, CYP2R1, CYP24A1, or VDR (especially polymorphs FokI, TaqI, ApaI, and BsmI), can affect the individual’s response to anti-infectious treatment, such as interferon/ribavirin therapy in chronic hepatitis C [13,14], susceptibility of the individuals to cancer, tuberculosis, ulcerative colitis, and Crohn’s disease, or the increased risk of type 1 diabetes, as was noted in European people [15,16,17,18].

### 2.3. Guidance Serum Concentrations

The most valuable indicator of the body’s vitamin D status is the serum levels of 25(OH)D, because of the relatively high affinity of that metabolite to DBP and its long serum half-life, ca. 25 days. Measurement of 1α,25(OH)_2_D to define vitamin D level in the organism is not recommended, as its serum half-life is only a few hours (ca. 7 h) [9,12]. Guidance serum concentration of 25(OH)D, which indicates an efficient level of vitamin D in the organism, is 30–80 ng/mL (ca. 75–200 nM/L). A severe vitamin D deficiency is reflected in serum concentrations below 10 ng/mL [12,19,20,21,22,23].

## 3. Vitamin D as an Anti-Infective Agent

The clear functions of vitamin D in the immune system are difficult to define because the immune response is not a static process and depends on the stage of infection.

The VDR, which has also been detected in immunological cells, suggests that vitamin D can regulate some processes related to immunity. As was shown in vitro, activated human T and B cells and also the endothelial cells lining the upper and lower respiratory tract can transform inactive metabolite 25(OH)D into active 1α,25(OH)_2_D. This compound acts on immune cells in an autocrine, paracrine, or intracrine way (i.e., throughout the pathways inside the cells) [24,25,26].

**Figure 2:**
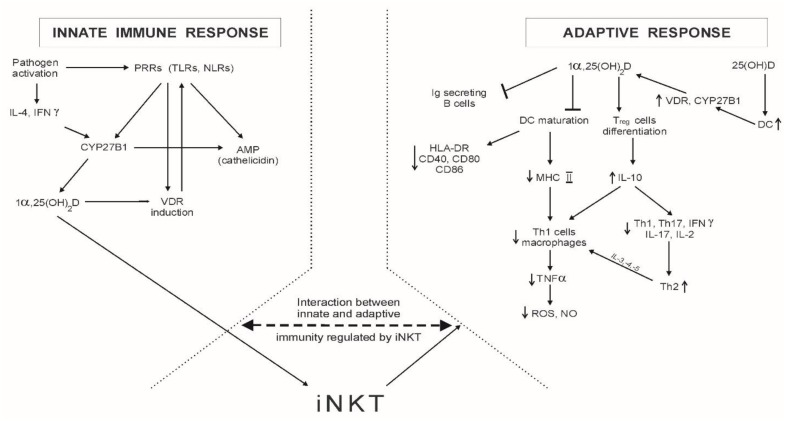
The role of vitamin D in the immune response. Abbreviations: PRRs (pathogen recognition receptors); TLRs (Toll-like receptors); NLRs (nucleotide-binding oligomerization domain (NOD)-like receptors); IL (interleukin); IFNγ (interferon γ); CYP27B1 (cytochrome P450-associated 25(OH)D(3)-1α-hydroxylase); AMP (antimicrobial peptides); VDR (vitamin D receptor); iNKT (invariant NK T cells); Ig (immunoglobulin); DC (dendritic cells); Treg cells (regulatory T cells); HLA-DR (human leukocyte antigens); CD (costimulatory molecules); MHC (major histocompatibility complex); Th cells (T-helper cells); IFNγ (interferon γ); ROS (reactive oxygen species); NO (nitric oxide).

The possible role of vitamin D in infectious diseases is implied by its impact on the innate and adaptive immune responses (Figure 2):

### 3.1. Vitamin D and the Innate Response

#### 3.1.1. Pathogen Recognition Receptors—PRRs

The innate immune response can be defined, generally, as nonspecific, although it proves to be the first line of defense against infective agents and initiates antigen presentation [27,28].

The crucial points for the innate immune response are the Toll-like receptors (TLRs), being a subgroup of various intracellular innate PRRs which is present in macrophages, polymorphonuclear cells, monocytes, and epithelial cells. TLRs recognize molecules related to the pathogen; for example, the lipopolysaccharides of bacteria or viral nucleic acids and proteins. Such activated TLRs release cytokines which induce reactive oxygen species and antimicrobial peptides (AMPs), cathelicidins, and defensins [4,6,7,24,25]. Several TLRs affect or are affected by VDR induction. For example, expression of the coreceptor for TLR4, costimulatory molecule CD-14, is induced by 1α,25(OH)_2_D in monocytes and epidermal keratinocytes. In turn, the increased expression of CYP27B1 in macrophages is the indirect result of AMPs, which stimulates TLR2 [6]. As shown by Greiller and Martineau [7], ligation of the TLR2/1 heterodimer in macrophages has been demonstrated to upregulate CYP27B1, similarly to the ligation of TLR8 by CL097 or TLR4 by lipopolysaccharide (LPS).

The exact mechanism of TLR ligation-induced CYP27B1 production is not fully understood, but it is possible that other TLRs or alternate PRRs may enhance extrarenal activation of that enzyme, allowing calcitriol to have more extensive effects on the immune response [7]. Upon viral infection, pathogen-associated molecular patterns (PAMPs) can also be recognized by other PRRs, such as retinoic-acid-inducible gene-I (RIG-I)-like receptors and nucleotide binding-oligomerisation domain (NOD)-like receptors (NLRs). In myeloid and epithelial cells, the intracellular receptor NOD2 is induced by 1α,25(OH)_2_D via two VDREs in the NOD2 gene. The addition of lysosomal breakdown products of bacterial peptidoglycan to calcitriol-induced NOD2 enhanced NFκB signalling and AMP such as beta defensin 2 expression [7,29].

The inflammatory cytokines TNFα and interleukins (IL) IL-1β, -6, and -12 are produced at an early stage of the innate immune response. These cytokines, among others, induce synthesis of acute phase proteins and contribute to the recruitment and activation of cells of the adaptive immune response. PRR signalling also results in the production of chemokine ligands (CXCLs), such as CXCL8–CXCL10 and IL-15, which generates neutrophils and natural killer cells (NK). These cells have an immune role, especially in bacterial innate immunity [7]. 

Upregulation of TLRs can also involve other mechanisms. Neutrophils, unlike macrophages, express the VDR, but they do not show an active 1α-hydroxylase. Thus, in these types of cells, it is not possible to induce transformation of 25(OH)D into the active metabolite upon TLR stimulation. In such cases, the surface proteins, such as the triggering receptor on myeloid cells-1 (TREM-1) or transforming growth factor β (TGFβ), present on the neutrophils or epithelial keratinocytes, respectively, may participate in the cell response to circulating 1α,25(OH)_2_D, including TLR signalling, via TREM-1, or by stimulation of CYP27B1 expression, via TGFβ [29]. TGFβ can cooperate with calcitriol to induce 5-lipooxygenase (5-LO), which catalyzes the synthesis of leukotrienes, compounds which participate, among others, in the phagocytosis of bacteria [29].

One of the features of the antibacterial innate response is the destruction of the pathogens by autophagy [26]. According to Chun et al. [29], recent data suggest that this process is important for the antibacterial response induced by vitamin D against *Mycobacterium tuberculosis* infection.

TLR-released AMPs have a broad spectrum of activity, not only microbial but also antiviral, and have been shown to inactivate the influenza virus [24]. The antiviral effects of AMPs are the result of, among other effects, the destruction of envelope proteins done by cathelicidins. Regarding antibacterial activity, AMPs induce, among other effects, membrane disruption. In humans, the active antimicrobial cathelicidin 37-residue, the amphipathic, helical peptide LL-37, is cleaved from the human cathelicidin propeptide (hCAP18). The majority of cathelicidin is stored in neutrophil granules, but also the other types of immune cells, as monocytes and NK and B lymphocytes can express hCAP18 [4]. Production of cathelicidins in human macrophages in vitro is stimulated by the active metabolite of vitamin D, 1α,25(OH)_2_D, via increased expression of the VDR [25]. According to Sundaram and Coleman [25], VDRE upregulation by TLRs leads to the transcription of cathelicidin, which kills intracellular *Mycobacterium tuberculosis*. As was shown by Szymczak and Pawliczak [26], hCAP18 also has activity against viruses and other bacteria. The lung epithelial cells, during viral infection, are capable of converting calcidiol into the active metabolite calcitriol, leading to increased hCAP18 production. As shown by Beard et al. [4], cathelicidin expression in macrophages and keratinocytes is induced by CYP27B1, and if there is no 25(OH)D, VDR, or CYP27B1, the ability of these cells to produce cathelicidins is significantly impaired. Following Szymczak and Pawliczak [26], not only TLR signaling, but also cytokines such as IL-4 and IFNγ may affect the CYP27B1 expression. The presence of IFNγ stimulates macrophage CYP27B1. It is also interesting that 1α,25(OH)_2_D participates in the negative feedback mechanism that self-inhibits the hyperactivation of TLRs [26].

It is worth noting that the impact of 1α,25(OH)_2_D on viral pattern recognition receptor-driven cytokine production varies between pathogens. As shown by Fitch et al. [30], in viral responses, it failed to modify TLR7/8- or respiratory syncytial virus (RSV)-stimulated innate cytokine production, even in supraphysiologic concentrations.

#### 3.1.2. Antimicrobial Peptides—AMPs

Vitamin D also regulates the other type of AMPs: defensins. Human beta defensin 2 is modestly stimulated by 1α,25(OH)_2_D and its antiviral effects arise from chemoattractive properties for neutrophils and monocytes [4,25]. However, serum 25(OH)D concentration was not associated with levels of serum AMPs in patients with community-acquired pneumonia [25].

Genome analysis revealed VDRE within the promoters of cathelicidin and beta defensin 2. Surprisingly, only cathelicidin appeared to be transcriptionally induced by 1α,25(OH)_2_D in monocytes [29]. As shown by the authors, the increased level of beta defensin 2 resulted from the decreased expression of 1α,25(OH)_2_D and interleukin-1 (IL-1) from monocytes, and that AMPs, for gene induction, require the cooperation of VDRE in the promoter and nuclear factor κB (NFκB) as the other transcription factor.

### 3.2. Vitamin D and the Adaptive Response

#### 3.2.1. T Lymphocytes

The basis of the adaptive response is: antigen presentation to B and T cells, and the antigen-stimulated production of antibodies and a wide spectrum of cytokines, chemokines, enzymes, and hormones. The initial observation related to the role of vitamin D in the immune system was the presence of the VDR in the activated lymphocytes [29].

T lymphocytes (T cells) include a few types of cells (called subgroups): CD8^+^ T cells, expressing relatively high levels VDR and vitamin D-activating 1α-hydroxylase; CD4^+^ T cells; NK cells; and memory cells. Activated CD8^+^ T cells can be differentiated into cytotoxic lymphocytes (CTLs), crucial for control against intracellular pathogens and cancer. Activated CD4^+^ T cells can be differentiated into T helper cells (Th cells), such as regulatory T cells (Treg) (called suppressor T cells), γδT cells, and Th17, Th9, Th1, and Th2 cells, which produce different profiles of cytokines. So, Th1 cells produce IL-2, TNFα, and interferon γ (IFNγ), and Th2 cells produce IL-3, -4, -5, -10, and -13. CD4^+^ T-induced cells, via cytokine secretion, provide support to other immune cells, such as CTLs, and to the serum antibody response, via CD40:CD40 ligand costimulation of antigen-specific B cells [6,25,26,27,29,31]. Besides cytokine production, the role of Th cells includes: the support of immunoglobulin production, macrophage activation, and production of eosinophiles and mastocytes (Th2) [27]. The Th1-stimulated response is the key for many bacterial and viral infections, but if it is an uncontrolled process, it leads to autoimmunity [28].

What is the role of vitamin D in that type of immune response? It acts as a modulator of Th cell proliferation and cytokine production, but also through promoting Treg cells, which are responsible for anti-infectious action, for suppressing immune responses, and for limiting inflammatory processes [28]. The exact mechanism is not well-known. One of the reports focused on the ability of 25(OH)D to stimulate Treg cells through induction of the antigen-presenting dendritic cells (DC) expressing VDR and CYP27B1 [32]. It is interesting to note that, as reported by Jeffrey et al. [32], the form of vitamin D significant for the generation of Treg cells was non-DBP-bound 25(OH)D. Bruce et al. [28] mentioned that 1α,25(OH)_2_D regulates invariant NK T cells (iNKT), which can act as regulatory cells and participate in the interaction between innate and adaptive immunity. Induction of iNKT has been shown to be protective, including against autoimmune diseases. In turn, Sigmundsdottir et al. [33] indicate that 1α,25(OH)_2_D-stimulated chemokine receptor 10 (CCR10) expression on T cells enables communication with other immune-competent cells, as it recognizes CCL27 secreted by keratinocytes throughout the organism. In vitro, 1α,25(OH)_2_D inhibits the expression of Th1 cytokines and stimulates Th2 cytokines. As shown previously, the other subgroup of Th cells, i.e., Th17 cells, secrete IL-17, playing a role in autoimmune processes [6,29]. Inhibition of Th1 cells was also noted in vivo, in mouse DC [25].

Inverse associations between vitamin D concentrations and disease activity in patients with inflammatory bowel disease, type 1 diabetes, multiple sclerosis, rheumatoid arthritis, or autoimmune thyroiditis have been noted [34].

1α,25(OH)_2_D appeared to limit autoimmune reactions, among other means, by inhibiting Th17 cell activity [35]. Apart from that, 1α,25(OH)_2_D can decrease the ability to present antigens by the inhibition of DC maturation. The result of the decrease is the diminished expression of the human leukocyte antigen HLA-DR and costimulatory molecules such as CD40, CD80, and CD86. 1α,25(OH)_2_D induces the differentiation of Treg cells, which induces IL-10 production. This cytokine is thought to inhibit IL-12. 1α,25(OH)_2_D-treated DC expressed less costimulatory MHC (major histocompatibility complex) II than intact cells [28]. Accordingly, Th1 cell and macrophage production is diminished, although the ability to induce Treg cells is maintained [6,25,28]. The induced IL-10 also suppresses Th1 and Th17 cells and thus production of IFNγ, IL-17, and IL-2, leading to immune tolerance [6]. The above events make Th2 cells predominant. As a result, the enhanced secretion of IL-4, -5, and -13 further suppresses Th1 cells. In human monocytes in vitro, 1α,25(OH)_2_D was shown as the inhibitor of Th1 cell-mediated cytokines and tumour necrosis factor α (TNFα), and in vivo in mice as the suppressor of the secretion and production of Th17 cells by downregulation of IL-23 and -6 [6,25,28]. As shown by Bruce et al. [28], treatment of naïve CD4^+^ Th cells during Th17 cells priming with 1α,25(OH)_2_D inhibits IL-17 production. The other mechanism of immunomodulation related to vitamin D is the impact on DC gene expression, which is independent of the differentiation of these cells [29]. As shown by Chun et al. [29], DC gene expression can be regulated by two major metabolites of vitamin D: 25(OH)D and 1α,25(OH)_2_D.

On the basis of animal studies, it was shown that the promotion of Th2 cells may have adverse effects on allergic diseases such as asthma atopic dermatitis through induction of the inflammatory processes [6]. The increased production of Th2 cytokines (IL-4, -5, -13), noted in the acute phase of atopic dermatitis, suppresses cathelicidin and increases susceptibility to infection. In the chronic phase of the disease, Th1 cells were predominant [6].

By inhibiting IFNγ, 1α,25(OH)_2_D inhibits the stimulation of reactive oxygen species and nitric oxide production. These effects, along with the suppression of IL-17 secretion, are responsible for the reduction of resistance to pathogens such as *Toxoplasma* and *Citrobacter* [6,36,37]. Ehrchen et al. [36] showed that VDR-knockout mice developed an altered Th1 response in *Leishmania major* infection, as indicated by the normal production of IFNγ by CD4^+^ and CD8^+^ Th cells. In turn, Rajapakse et al. [37] observed reduced IFNγ and IL-12 levels in *Toxoplasma gondii*-infected mice, indicating inhibition of Th1 cell activity. Following 1α,25(OH)_2_D treatment, reduced counts of CD4^+^ Th cells and splenocytes and the marked induction of apoptosis were also noted. Ryz et al. [38] showed the reduction of Th17 cells in *Citrobacter rodentium*-infected mice treated with 1α,25(OH)_2_D. According to the impaired Th17 response, a defect in the production of antimicrobial peptide REG3γ was reported.

1α,25(OH)_2_D impairs the antigen-presenting and T cell-stimulatory capacity of monocytes and macrophages, with a decrease in MHC II and CD40, -80, and -86. Suppression of IL-12 and IL-23, which are involved in Th1 differentiation, is due to the 1α,25(OH)_2_D-mediated NFκB activation [7,28].

It is worth noting that human T cell responses are regulated in the presence of DC and the inactive metabolite of vitamin D, i.e., 25(OH)D. As described by Jeffery et al. [32], CYP27B1 is induced in DC upon maturation with LPS or upon T cell contact, which results in the synthesis and release of 1α,25(OH)_2_D, which, in turn, affects T cell responses.

#### 3.2.2. B Lymphocytes

1α,25(OH)_2_D inhibits proliferation and acts as a proapoptotic agent in activated human B cells in vitro. Although it does not act on the production of these cells, it is thought to inhibit their differentiation [25]. As shown by Fang et al. [39] in mice, the immune protection induced by the influenza virus primary infection significantly relies on the presence of B lymphocytes.

Some suppressive effects of 1α,25(OH)_2_D were noted with reference to immunoglobulin (Ig)-secreting B cells. 1α,25(OH)_2_D was specifically able to inhibit the development of them after mitogenic stimulation [40]. The impact on Ig is not the only mechanism of B cell–vitamin D interaction. Other reports have also shown the regulation of B cells by 1α,25(OH)_2_D through IL-10 and CCR-10 [41]. As reported by Heine et al. [42], human B cells, upon activation by the receptor CD40 and IL-4 signals, show increased expression of the gene for the 25(OH)D 1α-hydroxylase CYP1α, followed by the production of significant amounts of 1α,25(OH)_2_D. 1α,25(OH)_2_D enhances IL-10 expression in B cells by the transcriptional activity of VDR or through modulation of calcium signalling. On the basis of these studies, it can be suggested that the inactive metabolite of vitamin D, 25(OH)D, can also modulate the immune response.

### 3.3. Anti-Inflammatory Action of Vitamin D

As reported by Penna et al. [43] in studies with use of the VDR agonist elocalcitol, this compound inhibited IL-17 and proinflammatory cytokine IFNγ secretion in prostate-draining lymph node T cells from elocalcitol-treated nonobese diabetic mice [43,44]. These results focus on the anti-inflammatory properties of vitamin D.

As such, this vitamin acts through the promotion of the feedback loop to prevent the overactivity of antibacterial processes and inflammatory events. Such action includes the downregulation of TLR2 and TLR4 on monocytes [25,29]. 1α,25(OH)_2_D has been reported to decrease proinflammatory chemokine production in human respiratory epithelial cells and downregulate proinflammatory cytokines such as IL-6, -8, and TNFα in many different cells in vitro [25]. As shown in human lymphocytes, the anti-inflammatory effect of vitamin D can be carried out in part through NFκB inhibition. This transcription factor regulates expression of the genes encoding the inflammatory proteins produced during infection, such as cytokines, chemokines, acute phase proteins, or inducible effector enzymes [6,25].

Vitamin D-modulated T-cell proliferation is a part of the mechanisms leading to anti-inflammatory responses via the increased proliferation of the subgroup of T cells, CD8α. These types of T cells, unlike CD8^+^ T cells, are not cytotoxic, but may play a role in suppressing gastroinflammation [29].

### 3.4. Time-Dependence of Immunomodulatory Effects of Vitamin D

The immunomodulatory effect of vitamin D is related to early or late phases of infection. The correlation between serum 25(OH)D levels and the levels of α_1_-antichymotrypsin, an acute-phase protein in patients with tuberculosis, was associated with the illness, but not with the initial, acute-phase response to infection [45]. Also, the different interactions of 1α,25(OH)_2_D with different mechanisms were reported to depend on time in human leukemia cells. Tse et al. [46] reported the time-dependent biphasic regulation of NFκB in HL-60 cells. After exposure of HL-60 cells to 1α,25(OH)_2_D, there was an early, ca. 4h suppression and a late, 8–72h-prolonged reactivation of that transcription factor. Alongside that stimulation, there was an upregulation of inflammatory and anti-apoptotic genes such as TNFα, IL-1β, and Bcl-xL [46]. Such an effect can indirectly impact the immune regulation shown by vitamin D because the inflammatory processes are strictly related to the immune response of the cells to pathogens. According to Sundaram and Coleman [25], the results of the study in vivo on the immune response to allergens in mice suggested that vitamin D supplementation (100 ng of 1α,25(OH)_2_D given as injection) given after the initial period of sensitization prevented high levels of eosinophils and the local inflammatory response in bronchoalveolar lavage fluid and lung tissues. Surprisingly, the lack of this effect was reported during supplementation every day during the study.

## 4. Vitamin D versus Influenza—Prevention or Therapy?

### 4.1. Anti-Infective Mechanisms of Vitamin D

The following ascertainment very accurately announces the possible relations between the main seasonal infection, i.e., influenza and vitamin D status in the body:

““Whoever wishes to investigate medicine properly should proceed thus: in the first place to consider the seasons of the year…” (Hippocrates, ca. 400 BC) [24].”

Vitamin D could be acknowledged as a “seasonal stimulus”, following R. Edgar Hope-Simpson, the British practitioner and self-educated epidemiologist. After documentation that influenza A epidemics in temperate latitudes are most intense in the months following the winter solstice, he hypothesized that solar radiation produces a “seasonal stimulus” that affects the pathogenesis of influenza A. He theorized that there is a seasonal steroid hormone system with an impact on the human immune system whose substrate levels are low during the influenza season, but peak when influenza is rare [24,47].

There are a few arguments which support vitamin D as a likely candidate for the abovementioned “seasonal stimulus”. In summary, most important is the 1α,25(OH)_2_D-stimulated production of AMPs, such as defensin and cathelicidin. As mentioned, these endogenous antibiotics act directly, destroying not only microbial pathogens, but also viruses, including the influenza virus [24,48,49]. The production of cathelicidin is dose-dependent on the serum level of 1α,25(OH)_2_D. As shown by Lang and Samaras [49], 30 ng/mL is necessary for the optimal induction of cathelicidin mRNA, but a higher level of 40 ng/mL was not more efficient. The next argument which supports the antiviral activity of vitamin D is the modulation of the inflammatory response as mentioned above. The release of proinflammatory cytokines by the influenza virus appeared to correlate with the severity of illness [24]. Khare et al. [50] noted that treatment of human lung A549 epithelial cells with 100 or 30 nM of 1α,25(OH)_2_D prior to or post-H1N1 (influenza A virus) exposure significantly decreased the levels of infection-induced TNFα, IFNβ, and IFN-stimulated gene-15 (ISG15) and downregulated IL-8 and IL-6 RNA levels. Following Helming et al. [51], 1α,25(OH)_2_D also potentially diminishes the proinflammatory cytokine production by the modulation of macrophages, which prevents them from the secretion of too many cyto- and chemokines. On the basis of the obtained data, the authors suggested that 1α,25(OH)_2_D participates in a negative feedback loop in which IFNγ-activated macrophages induce the release of 1α,25(OH)_2_D. When this metabolite is accumulated at an efficient concentration, VDR expression is synergistically induced and it translocates into the nucleus. Then, it can suppress the genes making the proinflammatory proteins, such as CCL5, CXCL16, IFI203, FCGR1, FCGR3, TLR2, IRF2, CXCL10, and CXCL9. Tight control of IFNγ responses is crucial for the consequences of granulomatous diseases such as tuberculosis and sarcoidosis [51].

### 4.2. Human Studies on Influenza Prevention by Vitamin D

A survey of the literature data generates some controversies and doubts about the possible role of vitamin D for the prevention of influenza infections. However, there are data obtained in vitro or in vivo which denote the antiviral activity of vitamin D in the case of influenza. Nowadays, the final conclusion is that its significance as an anti-influenza agent remains unresolved, but it does not mean that these considerations are senseless. It is most important to realise that the broad spectrum of vitamin D activity does not exclude such a role.

Some of the following studies do not strictly concern influenza infection, but also the influenza-like respiratory illnesses of respiratory tract infections (RIs) and pneumonia. However, the time period of the studies, i.e., October–March, or the winter months, does not exclude influenza infections, most common in the autumn and winter. According to Cannell et al. [24], if vitamin D is a “seasonal stimulus”, as has already been mentioned in this paper, then vitamin D deficiency should predispose patients to respiratory infections.

#### 4.2.1. Beneficial Effects

Moan et al. [52] compared the seasonality of deaths from influenza and pneumonia in Norway with vitamin D serum levels. The time period of the studies was 1980–2000. The final conclusion of these studies was that the high numbers of winter influenza and pneumonia deaths in Norway were related to low vitamin D levels in this season. The data support the hypothesis that vitamin D acts as a protector against influenza and pneumonia, although it is not clear if it requires any help, or which mechanism dominates in the battle against viral infections.

Laaksi et al. [53] conducted a placebo-controlled double-blind study (October–March) which involved 164 volunteering young Finnish men (18–28 years of age) undergoing military training. The subjects were randomly assigned to the intervention group (*n* = 80), which received 400 IU of vitamin D per day, or the placebo group (*n* = 84). After six months of the study, the supplemented group showed a mean serum concentration of 25(OH)D (±SD) of 71.6 ± 22.9 nM/L (*n* = 58) and the placebo group showed 51.3 ± 15.5 nM/L of 25(OH)D (*n* = 50) (*p* < 0.001). The main outcome considered was the number of days absent from duty due to respiratory infection. The proportion of men remaining healthy throughout the six-month study period was greater in the supplemented group (51.3%) as compared to the placebo group (35.5%) (*p* = 0.045). The above results provided some evidence for the preventive effect of vitamin D supplementation against respiratory tract infection, and according to the Cox regression analysis, the authors noted that the hazard ratio for absence from duty due to the respiratory tract infection was lower in the supplemented group as compared to the placebo group. As shown by the authors, randomized controlled trials with higher doses and larger populations are needed to explore the preventive effect of vitamin D supplementation on acute respiratory tract infection.

Promising and encouraging results on supplementation with vitamin D to prevent influenza were presented by Urashima et al. [54], who conducted a randomized, double-blind, placebo-controlled trial comparing vitamin D_3_ supplements (1200 IU/day) with placebo in schoolchildren from December to March. The outcome was the incidence of influenza A diagnosed with presence of the influenza antigen. As reported, influenza A occurred in 18 out of 167 (10.8%) children in the vitamin group compared with 31 out of 167 (18.6%) children in the placebo group (relative risk (RR) = 0.58; 95% CI: 0.34, 0.99; *p* = 0.04). The reduction of influenza incidence was greater in children who, among other factors, had not been taking other vitamin D supplements. It is interesting to note that in children with a previous diagnosis of asthma, asthma attacks as a secondary outcome occurred in two children receiving vitamin D compared with twelve children receiving placebo (RR = 0.17; 95% CI: 0.04, 0.73; *p* = 0.06). The above study, conducted up to 2012, was reported by Jorde et al. [55] as one properly performed randomized clinical trial on influenza prevention.

The positive role of vitamin D in respiratory infections and lung function was confirmed by Berry et al. [56], who used cross-sectional data from 6789 participants in a nationwide 1958 British birth cohort. The authors measured 25(OH)D, lung function, forced vital capacity, and respiratory infections from the age of 45 years. They showed a linear association between vitamin D status and seasonal infections and lung function. Each 10 nM/L increase in 25(OH)D was associated with a 7% lower risk of infection. Similar results were also obtained in other studies. Aregbesola et al. [57] investigated the risk of hospitalized pneumonia in an ageing general population in eastern Finland. On the basis of the study, the authors suggested an inverse effect of the serum 25(OH)D concentration on the risk of developing pneumonia. Jones et al. [58] examined 46 residual blood samples from adults and children, some of whom experienced influenza virus infections of the respiratory tract. Assays were performed for retinol binding protein (RBP), vitamin D, and antibody isotypes. Results showed that 44 samples exhibited RBP and/or vitamin D insufficiencies or deficiencies. Besides, vitamin D correlated with blood IgM and IgG3, while RBP correlated with IgG4 and IgA. It is known that vitamins A and D are critical for healthy immune responses at mucosal surfaces in mice. Especially, IgA is a first line of defense against mucosal pathogens. So, according to the authors, the results suggested that also in humans, there is a correlation between vitamin A and D levels and antibody profile. The authors suggest that vitamins may support the dendritic cell development necessary for antigen presentation; T-cell activation and homing; B-cell activation, division, and maturation; and/or the stabilization of differentiated antibody-producing cells. According to Jones et al. [58], these results recognize the need for further studies on the correction of vitamin supplementation, particularly at the time of respiratory virus vaccination, to improve vaccine efficacy and for protection against respiratory tract diseases.

Intriguing effects were presented by Mamani et al. [59] and Brance et al. [60] in their studies. Both studies showed an inverse correlation between the level of 25(OH)D and the severity of CAP (community-acquired pneumonia), which was defined as the CURB65 score (confusion, uremia, respiratory rate, low blood pressure, ≥65 years). In addition, according to Brance et al. [60], higher 25(OH)D concentrations were found to be correlated with lower CCI (Charlson comorbidity index). Nanri et al. [61], in a nested case-control study in a cohort of workers in four companies in Japan during the winter season, found lower influenza risk to be associated with vitamin D sufficiency (≥30 ng/mL), but only among unvaccinated participants. In a subgroup vaccinated earlier against influenza, serum 25(OH)D concentration did not correlate significantly with the incidence of physician-diagnosed influenza.

#### 4.2.2. Studies Showing No Relevant Effects

However, there is still one fly in the ointment. There are in vitro and in vivo data, as well as data resulting from human studies, which do not prove any significance of vitamin D supplementation in viral respiratory infections.

Gui et al. [62] showed the negative impact of 1α,25(OH)_2_D treatment on the innate immune response generated by the H9N2 infection in mice, especially at the later stage of the disease. Although it decreased the influenza M gene (encoding the M protein related to inflammatory response and virus replication), IL-6, and IFNβ in A549 cells prior to and post-infection with H9N2 influenza, the authors found that it did not affect virus replication in vitro and in vivo. Besides, the effect of 1α,25(OH)_2_D treatment was dependent on the stage of the illness. As shown in vivo, 1α,25(OH)_2_D downregulated pulmonary inflammation in mice two days post-infection, but increased the inflammatory response 4 to 6 days post-infection. Simultaneously, the expression of the antiviral cytokine IFNβ was significantly higher at two days post-infection and lower on days 4 and 8. These effects were consistent with the period of maximum body weight loss and the lung damage in calcitriol-treated mice. The reason for the positive anti-inflammatory activity of 1α,25(OH)_2_D noted in A549 cells and the opposite effect reported in mice during the later stage of infection is not clear. The authors indicate two possible explanations. The first is that the activity of vitamin D in vivo is complex and has an impact on so many pathways and mechanisms that it can affect one component of this system, but not the other. Secondly, avian influenza viruses such as H9N2 induce different mechanisms in mice and in humans. This is in agreement with the conclusion given by Grant and Giovanucci [48], who discussed, regarding the data showing that suppressing proinflammatory cytokines by vitamin D did not reduce the risk of death in mice infected with H5N1 viruses, that such an effect should not be applied to the H1N1 infections in humans because of the differences in immune response. The anti-inflammatory activity of 1α,25(OH)_2_D on A549 infected with H1N1 was also shown by Khare et al. [50]. The authors noted that 1α,25(OH)_2_D treatment prior to or post-infection downregulated IL-6 and IL-8 RNA levels and decreased the levels of infection-induced TNFα, IFNβ, and ISG15. 1α,25(OH)_2_D did not affect viral clearance, similarly to findings reported by Gui et al. [62], but reduced autophagy and restored increased apoptosis seen in the H1N1 infection back to its constitutive level.

In the studies presented by Jorde et al. [55], vitamin D appeared to make influenza infection a significantly more prolonged disease than in the patients receiving placebo. In the study, 569 subjects from 10 different clinical trials were included. Of the subjects, 289 were randomized to receive vitamin D (1111–6800 IU/day) and 280 received placebo. Influenza-like disease was reported in 38 subjects in the vitamin D group and 42 in the placebo group. In these groups, 25 and 26 subjects, respectively, showed clinical symptoms of influenza according to the defined criteria. In the vitamin D group, the duration of the illness was significantly longer than in the placebo group (2–60 days versus 2–18 days; *p* = 0.007). One of the weaknesses of this study, as emphasized by the authors, was that the study was retrospective and relied on self-reported symptoms; thus, there was not a definite diagnosis of influenza.

Some conflicting results were presented by Urashima et al. [63]. As was given by the authors, a randomized controlled trial on the effects of vitamin D supplementation on influenza illness during the 2009 H1N1 pandemic revealed that influenza A or B occurred less in the vitamin D group than in the placebo group only during the first half of the study. During the second month, the vitamin D group’s results were similar to those of the placebo group. The authors observed similar effects, i.e., preventive action of vitamin D supplementation only in the initial part of the studies, in the studies conducted among the students who received 2000 IU of vitamin D per day for two months. As shown by post-hoc analysis, influenza A occurred significantly less in the vitamin group (2/148, 1.4%) compared with the placebo group (8/99, 8.1%), but only in the first month of the study. The initial benefit was lost during the second month. These results could have been related with longer supplementation with vitamin D and are indirectly consistent with the results of Urashima et al. [54], who did not note the impact of vitamin D on influenza A incidence in children who had been taking more than one vitamin D supplement.

The lack of any correlation between respiratory infections and vitamin D supplementation was showed by Li-Ng et al. [64], who described a randomized controlled trial for the prevention of symptomatic upper respiratory tract infections, conducted during the winter. In total, 162 adults received 2000 IU of vitamin D per day for 12 weeks. There was no difference in the incidence of infections and in the duration or severity of respiratory tract infection symptoms between the supplemented and the placebo groups (48 vs. 50 cases, respectively, *p* = 0.57, and 5.4 ± 4.8 days vs. 5.3 ± 3.1 days, respectively, *p* = 0.86). It is worth noting that after 12 weeks, the mean serum concentration of 25(OH)D in the supplemented group was 88.5 ± 23.2 nM/L and in the placebo group was 63.0 ± 25.8 nM/L. As a matter of fact, the serum concentration of 25(OH)D in the supplemented group was not too efficient; the value 88.5 ± 23.2 nM/L is placed near the defined lower limit of the 25(OH)D level reported as the guidance level (75–200 nM/L) [12]. In studies conducted in 2007, the same authors found a significant reduction in colds and influenza in women taking 800 or 2000 IU/day of vitamin D. Surprisingly, in the final year of the study, women supplemented with 2000 IU were still vitamin D-deficient. [65]. According to Aloia and Li-Ng [65], the trial should use enough cholecalciferol to raise 25(OH)D levels to those achieved by natural skin synthesis in the summer, i.e., ca. 50 ng/mL. A similar lack of protective effects of vitamin D was found by Lappe et al. [66] in double-blind, placebo-controlled, population-based, randomized clinical trials. Among healthy postmenopausal older women with a mean baseline serum 25(OH)D level of 32.8 ng/mL, supplementation with vitamin D and calcium compared with placebo did not result in a significantly lower risk of all types of cancer after four years.

#### 4.2.3. Critical Views

##### Studies on the Role of Vitamin D Supplementation

A systematic review and meta-analysis done by Martineau et al. [67] pointed to the issue which is most crucial for studies based on vitamin D supplementation. The authors presenting the results of 26 eligible randomized controlled trials showed that vitamin D supplementation significantly reduced the risk of acute respiratory tract infection (RI), but the protective effects were observed in those receiving daily or weekly vitamin D without additional bolus, and not in those receiving one or more bolus doses. The beneficial effects were negatively correlated with baseline 25(OH)D levels < 25 nM/L. In addition, the authors found that baseline vitamin D status and dosing frequency independently modified the effect of vitamin D supplementation on the risk of acute RI. On the basis of the results, the authors suggested that high doses of vitamin D supplemented as bolus firstly can evoke some adverse effects in circulating vitamin D metabolites. Secondly, the high concentrations after bolus may dysregulate the activity of the enzymes responsible for the synthesis and degradation of 1α,25(OH)D, which results in decreased concentrations of it in extrarenal tissues. In turn, the correlation is such: the stronger the effect, the lower the vitamin D level may be, according to the authors, based on the principle that people who are most deficient in a micronutrient will be the most likely to respond to its replacement [67]. This may explain the results obtained by Aloia and Li-Ng in 2007, described above, that showed that supplementation in women with a vitamin D-deficit was more protective against colds and influenza than in the case of vitamin D-sufficient women [65].

On the basis of the studies mentioned above, in which supplementation with vitamin D and the analogous higher serum concentration of this vitamin did not give the expected effect, Grant et al. [68] proposed a new approach to vitamin D randomized controlled trials to provide true confirmation of the role of its vitamin dosing being solely to achieve the targets set for achieved 25(OH)D concentrations. As shown by the authors, the assumption that the vitamin D dose–response relationship is linear is not true. The supplemented vitamin D has no direct health effect, because the conversion to 25(OH)D varies from individual to individual regarding genetic polymorphisms of CYP enzymes, intestinal absorption, or body mass. Grant et al. [68] proposed a design strategy which targets supplementation to the chosen baseline status while ensuring achievement of the desired status. This could be done through checking 25(OH)D concentrations periodically during the trial, as well as the baseline, and through recruiting nonreplete subjects. With such an approach, randomized controlled trials would have an increased potential for detecting causality.

##### Seasonality of Vitamin D Levels and Influenza Rate

As shown by Shaman et al. [69], seasonal variation in the serum concentration of 25(OH)D, which contributes to immune function, has been hypothesized to be the underlying source of observed influenza seasonality in temperate regions. So, the authors studied whether 25(OH)D levels could be used to simulate influenza infection rates. The studies were done in two regions of the United States. On the basis of best-fitting simulations which could reproduce the observed seasonal cycle of influenza, the authors concluded that it is unlikely that seasonal variations in vitamin D levels determine the seasonality of influenza in temperate regions. These results suggest that influenza transmission may be dependent on different factors. On the basis of a reanalysis of laboratory experiments, Shaman et al. [70] revealed that absolute humidity strongly modulates the airborne survival and transmission of the influenza virus. Also, Koep et al. [71] studied the impact of absolute humidity in the indoor school environment on virus survival. As noticed, classroom humidification may be a feasible approach to increase indoor absolute humidity to levels that may decrease influenza virus survival and transmission. In turn, Yang et al. [72] studied other meteorological factors which influence the dynamics of influenza in tropical Africa. They computed the monthly viral positive rate for three circulating influenza subtypes: A/H1N1, A/H3N2, and B, among patients presenting influenza-like illness or severe acute RI in two Ugandan cities. The impact of temperature, relative and absolute humidity, and precipitation, as well as interactions among the above influenza subtypes on the epidemic dynamics of each influenza subtype were studied. As observed, the associations with weather variables differed by influenza subtype. The models showed that precipitation and temperature were negatively correlated with A/H1N1 activity. A mutually negative association between A/H3N2 and B activity was identified.

Kroll et al. [73] mentioned the other factor conjugated with the level of 25(OH)D and parathyroid hormone (PTH) concentrations, which are known to have a reciprocal seasonal relationship with 25(OH)D. These two compounds vary in sinusoidal pattern throughout the year, even in ergocalciferol-treated patients. This means that 25(OH)D is higher in the summer and lower in winter, while PTH shows the reverse pattern. According to the authors, in these observations, held across three latitudinal regions, both genders and multiple years are applicable for patient care.

Independently of the results of the conducted studies, which support or do not support the positive role of vitamin D serum levels and supplementation in the prevention against influenza infections with influenza-related diseases, such as RI and pneumonia, most of the authors agree that this topic deserves further study. The questions: “What dose of vitamin D ensures more resistance to influenza infections?” and “Does vitamin D-deficiency mean more susceptibility to influenza infections?” remain open and require more clinical trials.

A summary of the results obtained in the human studies on the role of vitamin D in the upper respiratory tract infections and defined influenza can be found in Table 1.

## 5. Does Vitamin D Affect the Immunogenicity of Influenza Vaccines?

### 5.1. Vitamin D as an Adjuvant

According to annual reports, the influenza virus causes 3–5 million severe cases which result in 250,000 to 350,000 deaths worldwide [49]. Vaccination is the most important preventive strategy against influenza, with the crucial expected outcome being antibody production, although it is still not sufficiently effective to reduce the morbidity and mortality associated with influenza in humans [74]. As shown in this paper, vitamin D undoubtedly plays the role of an immunomodulating agent. Its significance in innate and adaptive immunity prompted scientists to check if there is any influence of vitamin D on the response of the patients to immunization by vaccination with live or inactive attenuated influenza virus, especially because some studies on influenza prevention provide a negative correlation between enhanced post-vaccine immunization and obese patients, whereas obesity implies vitamin D deficits [75,76]. These observations indirectly point to the possible role of vitamin D improvement of the immune response to influenza vaccines. Principi et al. [77] mentioned animal studies in which the results encourage regarding vitamin D as a kind of adjuvant agent for better influenza vaccine efficacy. The studies showed that the addition of vitamin D or 1α,25(OH)_2_D to a variety of vaccine preparations could increase induced immunity to the herpes simplex virus, diphtheria toxoid, tetanus toxoid, hepatitis B surface antigen, poliovirus, or HIVgp160. Following Sadarangani et al. [75,78], mature adult mice that were immunized subcutaneously or intramuscularly with inactivated polio vaccine, *Haemophilus influenzae* type b oligosaccharide conjugated to diphtheria toxoid vaccine, and hepatitis B surface antigen (HBsAg), coadministered with 1α,25(OH)_2_D, demonstrated production of antigen-specific mucosal immunity (IgA and IgG antibodies) as well as enhanced systemic immune response. As demonstrated by Lang and Samaras [49], vitamin D coadministered with trivalent influenza vaccine (TIV) in mice was shown to enhance the anti-hemagglutinin antibody response and mucosal immunity.

Even though vitamin D has profound effects on immunity, there are insufficient data to show the real relationship between vitamin D status and influenza vaccine immunization [45]. Sadarangani et al. [75,78] suggest that studying the impact of VDR-related polymorphism can provide further insight into the complex interactions in the context of vaccine-induced immune response.

As mentioned earlier, according to Jones et al. [58], the vitamins which support vaccine efficacy should be supplemented, particularly at the time of respiratory virus vaccination, to improve antigen presentation and antibody profile, and this warrants further studies.

### 5.2. Human Studies on the Impact of Vitamin D on Immunization

#### 5.2.1. Beneficial Effects

The general target groups for influenza vaccination are children and older adults. Following Principi et al. [77], influenza is common in pediatrics. Children are the main cause of the spread of the infection, and because of the immaturity of the immune system at ages less than five years, they can develop severe disease. The other target group, older persons, have impaired immune responses to influenza vaccination secondary to immunosenescence, inflammaging, and impaired T-cell responses or loss of dendritic cell function [25,75]. Additionally, this group of the population is more often exposed to vitamin D deficit because of decreased skin synthesis and decreased renal production of the active metabolite, 1α,25(OH)_2_D [75].

Literature data provide evidence both for and against the beneficial effect of vitamin D supplementation on antibody production, and the possible reasons for the negative results obtained are broadly discussed.

The most promising results were obtained by Chadha et al. [79] in prostate cancer patients. Response to the trivalent influenza vaccine, Fluzone, for the period 2006–2007 in 28 of 35 participants was defined as the increase in antibody titer at three months against all three strains of the virus positively correlated with 25(OH)D level, which was tested as a continuous variable in relation to serological response (*p* = 0.0446). The median baseline 25(OH)D level was 44.88 ng/mL (range: 9.16–71.98 ng/mL). This outcome was also evaluated in studies on adults aged above 50 years by Sundaram et al. [80], but the results obtained by the authors were opposite. Prospective cohort studies conducted during two influenza seasons (2008–2009) showed that more than 25% of the 1103 participants were vitamin D-deficient (<25 ng/mL), and the deficiency was associated with a greater frequency of post-vaccine seroprotection for the H1N1 virus, but only in the first year of the study. It was not related to seroprotection or seroconversion for any other strain in either year. Therefore, no consistent association was found between vitamin D levels and serological response to influenza vaccination in older adults.

Sadarangani et al. [75] reported a weak positive correlation between 25(OH)D levels and change in influenza-specific granzyme B response on day 75 post-vaccination (*p* = 0.04) in a cohort of 159 healthy subjects (50–74 years old) vaccinated with one dose of trivalent 2010–2011 influenza vaccine containing A/California/H1N1-like virus. The range of 25(OH)D defined in the studied group was from 36.6 to 52.2 ng/mL. These results do not exclude the role of vitamin D in improving immunization. Granzyme B is a serine protease produced by NK cells, dendritic cells, and cytotoxic T cells. It is known to induce the cytotoxic T-cell-mediated apoptosis of virus-infected host cells and has been evaluated as a promising marker of immunity response to the influenza vaccine. It is worth noting is that granzyme B response negatively correlates with increasing age [75]. In the same study, no correlation between 25(OH)D levels and humoral immune response was detected at any time point after vaccination.

#### 5.2.2. Studies Showing No Relevant Effects

No association between vitamin D and post-vaccine serological response was shown in HIV-infected patients vaccinated with monovalent influenza A (H1N1) by Crum-Cianflone et al. [81]. During a prospective cohort study of 124 participants (64 HIV-infected and 64 uninfected), seroconversion measured as >4-fold increase in antibody titer was achieved on day 28 post-vaccination in 56% of the HIV-infected persons versus 74% of those HIV-uninfected, but vitamin D deficiency was insignificantly different between both groups.

Studies on the immunization of children vaccinated with live attenuated or inactivated influenza vaccines in dependence of the 25(OH)D serum concentration conducted by Lin et al. [82] also did not bring an answer to the question: “Can vitamin D act as an adjuvant in influenza vaccines?”. Vitamin D levels in serum and influenza antibody titers were measured prior to and 21 days post-vaccination with live attenuated or inactivated influenza vaccine. Surprisingly, low vitamin D levels were associated with higher antibody titer against live attenuated virus, and this effect was strain-specific (*p* < 0.05). Similar results were obtained by Principi et al. [77] four years earlier. On the basis of a prospective, randomized, single-blind, placebo-controlled study with 116 children, the authors indicated that daily supplementation with 1000 IU of vitamin D for four months starting from the injection of the first dose of the trivalent influenza vaccine Fluarix did not significantly modify the antibody response. Similarly, Lee et al. [74] observed no correlation between 25(OH)D levels and post-vaccination antibody titer in a retrospective observational study conducted among 437 young healthy members of the military. Only 224 of them (51.3%) demonstrated an increase in anti-influenza post-vaccination titer, which was not associated with 25(OH)D levels.

A summary of the results obtained by some authors in vitro or in vivo or in clinical controlled studies on the impact of vitamin D on the serological response to anti-influenza vaccines can be found in Table 2.

#### 5.2.3. Critical Views

The authors have a critical approach to the clinical studies which do not prove a beneficial effect of vitamin D on the immune system or indicate the lack of any effect of this vitamin on post-vaccine immunization. The main potential reasons for this outcome which are most often considered are: too short a time between vaccination and antibody assays, which may be not sufficient to reveal immunostimulation; or too low a dose of vitamin D, giving too low a concentration level of 25(OH)D to mediate in immunization processes [49]. For example, Lee et al. [74], in their studies, used the clinical cutoffs for insufficiency of 20–30 ng/mL and for deficiency of <20 ng/mL, while as demonstrated by the authors, some experts have shown that a higher level is required (≥40 ng/mL). Some authors also admit that in their studies, T cells were not included. Therefore, any potential impact of vitamin D on this part of the immune response has not been explored [74,80]. The next potential reason is too-small samples being used in the studies or the profiles of the immune system in the patients with comorbid diseases being different than in the healthy subjects, which can potentially determine the effect of vitamin D on the response to the influenza antigen, as observed by Chadha et al. [79]. Besides, studies such as the above should be conducted on a group which is representative for other populations [74]. As Lang and Samaras [49] mentioned, for example, the antibody level present in the preimmunized serum can mask the potential immunomodulatory effect of vitamin D on the immune response post-vaccination because of the inverse relationship between antibody levels prior to and antibody increase post-vaccination. Few studies have included subjects already on vitamin D supplementation, or they were conducted in selected populations [49,74,75,78].

The work described and done by Surman et al. [83] raised the very important question of the interaction of different factors resulting in the immune response. The authors reported in mice immunized with an attenuated influenza virus vaccine that double deficiencies for vitamin A and D reduced antibody response in the respiratory tract to a greater extent than deficiency for one of these vitamins. Although supplementation with vitamin A had a greater corrective effect than vitamin D for the restitution of seroprotection (IgG and IgA responses), the best results were with the two vitamins combined and administered at the time of the vaccination of the animals. These studies, although they are animal-based, give some useful information for designing human clinical trials for the improvement of influenza vaccine efficacy and show that the approach to the significance and the potential role of supplementation with micronutrients, such as vitamins, to reach this aim should regard their interaction and synergism of action [74,83].

Lee et al. [74] suggested the real role of vitamin D in immunization against influenza viruses. It is known that vitamin D supports anti-inflammatory processes through its impact on T cells. Hence, according to the authors, a measure of increased immunity may not be the mechanism of action by which vitamin D functions. It is possible that its higher level reduces the severity of the inflammatory response brought on by infections.

So, the presented results are not too enthusiastic regarding the improvement of influenza vaccine efficacy by vitamin D. However, in light of its fantastic properties, it is undoubtedly the only vitamin characterized by such a broad spectrum of activity in the immune system.

## 6. Summary

The survey of the literature concerning the role of vitamin D in the immune system and immunization, especially against influenza viruses, does not give an unequivocal and one-word answer of “yes” or “no” to the questions: “Does vitamin D supplementation enhance the host’s resistance to influenza?” and “Does vitamin D supplementation play a role in the therapy of viral infection diseases?”. The authors, as presented, despite the lack of the expected results, do not exclude the significance of this vitamin for the anti-influenza battle waged in human organisms. Some of them suggest that the effects of 1α,25(OH)_2_D on chemokine expression and secretion vary between pathogens [7]. The results of the presented studies also do not exclude the premises to including vitamin D as an adjuvant in directing new influenza vaccine design. Following Wiwanitkit [84], in spite of the unconfirmed usefulness of vitamin D as an adjuvant for influenza vaccination, giving it simultaneously still poses some clinical advantages. According to Grant and Giovanucci [48], vitamin D supplements or fortified foods should be evaluated further as a possibly useful component of a programme to reduce influenza mortality rates, especially in elderly persons.

The studies clearly show that vitamin D is, undoubtedly, part of the complex factors which affect the immune response. So, assessing vitamin D status and maintaining optimal serum levels should be considered in all ageing adults and children, and micronutrients should be regarded as one of the essential factors which improve our health condition overall and also support our fight against diseases.

## Figures and Tables

**Table 1:** The selective human studies on the effect of vitamin D supplementation on influenza, upper respiratory tract infections (URI or RI), and pneumonia incidence published between the years 2009 and 2018.

Author, Year	Trial/Duration/Location	Sample Size/Participants	Vitamin D Dose Supplemented per Day	Outcome Measure	Result	Statistical Significance
Li-Ng et al., 2009 [64]	Randomized controlled study/winter season/USA	162/adults	2000 IU	Primary outcome: incidence of URI symptoms on the basis of biweekly questionnaire	48 cases of URI symptoms in the supplemented group vs. 50 cases of URI symptoms in the placebo group	*p* = 0.57
				Secondary outcome: duration and severity of URI symptoms on the basis of biweekly questionnaire	5.4 ± 4.8 days in the vitamin group vs. 5.3 ± 3.1 days in the placebo group	95% CI: −1.8 to 2.1 *p* = 0.86
Laaksi et al., 2010 [53]	Randomized, double-blind, placebo-controlled study/Oct–March/Finland	164/men 18–28 years old, undergoing military training	400 IU	The number of days absent from duty due to URI	51.3% of men remaining healthy in the intervention group vs 35.5% in the placebo group	*p *= 0.045
Urashima et al., 2010 [54]	Multicenter, randomized, double-blind, placebo-controlled parallel-group study/Dec–March/Japan	167/schoolchildren	1200 IU	Primary outcome: influenza A antigen vs incidence of influenza A*	18/167 (10.8%) children in the suppl. group vs 31/167 (18.6%) children in the placebo group	RR = 0.58; 95% CI: 0.34, 0.99; *p *= 0.04
				Secondary outcome: asthma attacks in the case of a previous diagnosis of asthma	Asthma attack incidence in 2 children in the suppl. group vs 12 children in the placebo group	RR = 0.17; 95% CI: 0.04, 0.73; *p* = 0.06
Berry et al., 2011 [57]	Cross-sectional study/Great Britain	6789/1958 British birth cohort aged above 45 years	-	25(OH)D serum concentration vs. RI	Each 10 nM/L increase in 25(OH)D was associated with a 7% lower risk of RI	95% CI: 3.11%
				25(OH)D serum concentration/lung function vs forced expiratory volume in 1 s (FEV1) and forced vital capacity (FVC)	Each 10 nM/L increase in 25(OH)D was associated with 8 mL higher volume of FEV1 and 13 mL higher volume of FVC	95% CI: 3, 13 for FEV1 95% CI: 7, 20 for FVC
Jorde et al., 2012 [55]	Retrospective study in subjects participating in randomized, double-blind, placebo-controlled studies/fall–winter 2010/Norway	569/men and females aged 32–84 years from 10 different clinical trials	1111–6800 IU	Primary outcome: influenza-like diseases and influenza reported on the basis of the questionnaire	25/289 subjects receiving vitamin D and 26/280 subjects receiving placebo were reported as infected with defined influenza	*p* = 0.064
				Secondary outcome: Duration and severity of the illness	The median duration of the illness in 24/25 subjects infected with influenza was 7 days and in the placebo group was 4 days.	*p* = 0.007
Aregbesola et al., 2013 [56]	Prospective study/9.8 years/Finland	1421/723 men and 698 women, 53–73 years old	-	25(OH)D serum concentration vs. the risk of incident hospitalized pneumonia	The subjects in the lowest 25(OH)D serum conc. tertile had a 2.6-fold higher risk of developing pneumonia with the subjects in the highest tertile	95% CI: 1.4 to 5.0; *p* = 0.05
Jones et al., 2015 [58]	Laboratory study/USA	46 blood samples/adults and children	-	Retinol binding protein (RBP) vs. antibody isotypes	44 samples exhibited the correlated RBP and/or vitamin D insufficiency or deficiency as follows: RBP <22,000 ng/mL, 25(OH)D <30 ng/mL RBP correlated with:	R_S_ = 0.31, *p* = 0.04
					IgA	R_S_ = 0.47, *p* = 0.0009
					IgA/IgM	R_S_ = 0.33, *p* = 0.03
					IgA/IgG1	R_S_ = 0.51, *p* = 0.0003
					IgG4	R_S_ = 0.39, *p* = 0.0082
				25(OH)D concentration vs antibody isotypes	25(OH)D concentration correlated with:	
					IgM	R_S_ = 0.36, *p* = 0.01
					IgG3	R_S_ = 0.32, *p* = 0.03
					IgG3/IgG1	R_S_ = 0.36, *p* = 0.01
					IgG3/IgA	R_S_ = 0.31, *p* = 0.04
Mamani et al., 2017 [59]	Case-control study/Iran	73 patients with CAP, 76 healthy controls	-	25(OH)D serum concentration vs. the risk of the incidence of CAP	The risk of pneumonia among the subjects with 25(OH)D <10 ng/mL was 3.69	95% CI: 1.46, 9.31; *p* = 0.006
Brance et al., 2018 [60]	Observational study/July 2015–June 2016/Argentina	167 patients with CAP, 59% women, 57.4 ± 19.6 years old	-	25(OH)D serum concentration vs. CURB65 score and CCI	25(OH)D serum concentration inversely correlated with the severity of	
					CAP (CURB65 score)	*p* = 0.049
					and with CCI	*p* = 0.07

RR—Relative risk; R_S_—Spearman’s rank correlation coefficient; CI—Confidence interval; CAP—community-acquired pneumonia; CCI—Charlson comorbidity index.

**Table 2:** Results of human studies on the association between vitamin D serum level and the serological response to anti-influenza vaccines published between the years 2011 and 2018.

Author, Year	Trial/Location	Sample Size/participants	Anti-Influenza Vaccine/Vitamin D Supplemented per Day	Outcome Measure	Time Points	Results	Statistical Significance
Chadha et al., 2011 [79]	Prospective study/USA	35/prostate cancer patients	Fluzone 2006–2007 trivalent vaccine containing: A/New Caledonia/20/99(H1N1), A/Wisconsin/67/2005(H3N2), B/Malaysia/2506/2004 viruses/-	Serum 25(OH)D conc. vs. antibody titer with HAI	3 months post-vaccination *	28 of 35 subjects showed 4-fold antibody titer increase against all three strains correlated with serum 25(OH)D conc. in the range: 9.16–71.98 ng/mL	*p* = 0.0446
Sundaram et al., 2013 [80]	Prospective, cohort study/USA	1103/adult volunteers aged ≥50 years	Trivalent vaccines containing:	Serum 25(OH)D conc. vs.	Pre-vaccination and 21–28 days post-vaccination in two seasons: fall 2008–spring 2009 and fall 2009–spring 2010	≥4-fold rise in HAI to post-vaccination against H1N1 strain associated with vitamin D deficiency, i.e.,	OR = 1.68, 95% CI = 1.13–2.49
			Season 1: A/Brisbane/59/2009-like(H1N1), A/Brisbane/10/2007-like(H3N2), B/Florida/4/2006-like viruses	antibody titer with HAI		serum 25(OH)D concentration <25 ng during season 1	
			Season 2:				
			A/Brisbane/59/2009-like(H1N1),				
			A/Brisbane/10/2007-like(H3N2), B/Brisbane/60/2008-like, influenza A(H1N1)pdm09-like[A(H1N1)pdm09]/-				
Principi et al., 2013 [77]	Prospective, randomized, single-blinded, placebo-controlled study/Italy	116/children with a history of recurrent acute otitis media previously unvaccinated	Fluarix, trivalent vaccine containing: A/California/7/2009(H1N1)-like, A/Perth/16/2009(H3N2)-like, B/Brisbane/60/2008(B)-like viruses/1000 IU for 4 months	Antibody titer with HAI vs. vitamin D supplementation	3 months post-vaccination	No significant correlation	No significance
Sadarangani et al., 2016 [75]	Retrospective, cohort study/USA	159/healthy subjects aged 50–74	Fluarix 2010–2011, trivalent vaccine containing: A/H1N1/California/2009-like virus/-	Antibody titer with VNA or HAI vs. serum 25(OH)D conc.	0, 28, and 75 days post-vaccination	No significant correlation	No significance
Crum-Cianflone et al., 2016 [81]	Prospective cohort study/USA	128/adults, 64 HIV-infected and 64 HIV-uninfected	Monovalent 2009 influenza A (H1N1) vaccine containing A/California/7/2009(H1N1)/ 2009–2010/-	Seroconversion vs. HIV infection	0 days, 28 days, and 6 months post-vaccination	Seroconversion at 28 days post-vaccination in 56% of HIV-infected patients vs. 74% HIV-uninfected persons.	*p* = 0.03
				Serum 25(OH)D conc. vs. HIV infection		Vitamin D deficiency was not significantly prevalent in HIV-infected patients (25%) compared to HIV-uninfected persons (17%)	*p* = 0.39
				Serum 25(OH)D conc. vs. seroconversion		No associations between serum 25(OH)D conc. and antibody responses at 28 days and after 6 months	No significance
Lin et al., 2017 [82]	Prospective cohort study/USA	135/children aged 3–17 years	Live attenuated influenza vaccine, LAIV B lineages (B Brisbane and B Massachusetts) and LAIV A strains (A/H1N1 and A/H3N2) or inactivated influenza vaccine, IIV/-	Serum 25(OH)D conc. vs. antibody titer with HAI	Pre-vaccination and 21 days post-vaccination	Serum 25(OH)D conc. were >20 ng/mL in 55% of persons.	*p* < 0.05
						Post-vaccination antibody titers for LAIV B were higher among those with lower 25(OH)D levels and among younger participants;	*p* < 0.05
						no associations between 25(OH)D conc. and responses to LAIV A strains or to any IIV strains	
Lee et al., 2018 [74]	Retrospective, cross-sectional observational study/USA	437/young healthy military members	Monovalent influenza A (H1N1) vaccine 2009 (strain A/California/7/2009/H1N1)	Immunogenicity vs. serum 25(OH)D conc. (seroprotection was defined as antibody titer ≥ 1:40 with MN	At least 30 days post-vaccination	34.8% of participants were vitamin D-deficient, 38.2% were insufficient, and 27.0% were normal in regard to serum 25(OH)D conc.; 51.4% of total participants showed seroprotection	Geometric mean titer: insufficient vs. normal: OR 1.25 (0.78–2.01); deficient vs normal: OR 1.10 (0.68–1.78); continuous 25(OH)D conc.: OR 0.98 (0.84–1.15)
						No associations between antibody response and any baseline characteristic	95% CI, *p* > 0.05

HAI—hemagglutination antibody inhibition assay; OR—odd ratios; VNA—viral neutralization assay; MN—microneutralization test.
